# Study of efficacy and safety of Jiaotai pill in the treatment of depression

**DOI:** 10.1097/MD.0000000000019999

**Published:** 2020-05-01

**Authors:** Zhihuan Zhou, Shufei Fu, Yijia Liu, Yuhan Wang, Huaien Bu, Yan Mei, Yi Tong, Chunquan Yu

**Affiliations:** a College of Traditional Chinese Medicine; b Graduate School, Tianjin University of Traditional Chinese Medicine; c Department of Psychosomatic Disease, Tianjin Hospital of ITCWM Nankai Hospital; d Department of Psychosomatic Disease, Tianjin Academy of Traditional Chinese Medicine Affiliated Hospital; e Clinical Department, First Teaching Hospital of Tianjin University of Traditional Chinese Medicine; f Editorial Department, Tianjin University of Traditional Chinese Medicine, Tianjin, China.

**Keywords:** depression, functional magnetic resonance imaging, Hamilton depression scale, Jiaotai pill, traditional Chinese medicine

## Abstract

**Background:**

Depression is a common affective disorder characterized by marked and lasting melancholia, with corresponding thought and behavior changes. Due to an accelerated pace of life and increased work pressure, the incidence of depression has risen sharply, causing great harm to family and social life. Jiaotai pill (JTP) is a Chinese herbal formula that is commonly prescribed for depression and insomnia in clinical treatment, and exhibits antidepressant effects as shown in animal experimental research. However, there are no standard clinical trials to confirm its efficacy in treating depression.

**Objective:**

This study aims to assess the efficacy and safety of JTP in the treatment of depression, so as to tap the clinical efficacy advantages of JTP and provide data support for its clinical application.

**Methods:**

A randomized, multicenter clinical trial with parallel groups was designed in this study. A total of 40 patients with depression were included and randomly divided to either the treatment or the control group with a ratio of 1:1. The patients received JTP plus fluoxetine or fluoxetine alone once per day for 8 weeks. The primary outcome included the Hamilton Depression Rating Scale score for patients and brain structure and function by functional magnetic resonance imaging. The secondary outcomes included Traditional Chinese medicine syndrome integral scale scores, Wisconsin Card Sorting Test, blood metabonomics, urine metabonomics.

**Conclusion:**

The results of this trial will find changes in brain structure, brain function, and metabolism in patients with depression, and provide critical evidence for JTP in the treatment of depression.

## 1 Introduction

Depression is a common mood disorder with high incidence rates of recurrence, disability and suicide.^[1]^ The World Psychiatric Association survey has shown that current incidences of depression are 4.2% globally and 6.9% in China, respectively. Depression has now become a serious worldwide public health and social issue to affect patients’ physical and mental health and reduce the quality of life. Moreover, depression can even lead to aggressive behaviors, and cause harm to individuals and families.^[2–5]^

**Figure 1:**
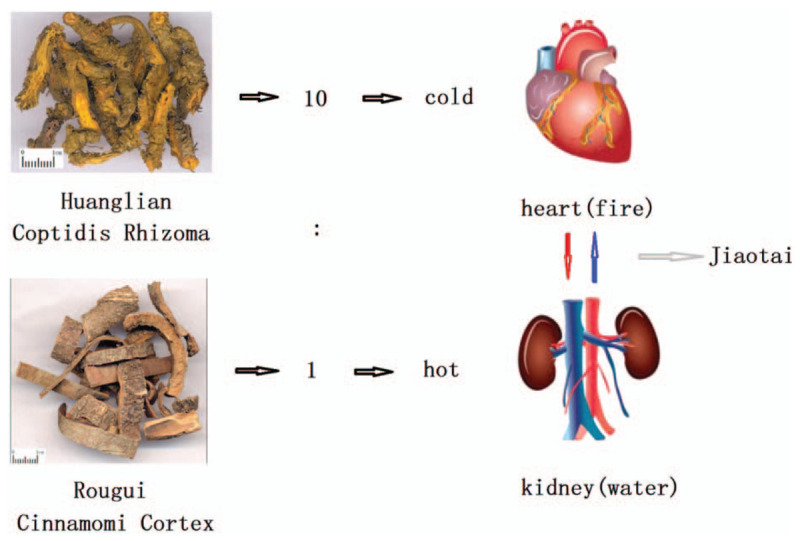
The composition of Jiaotai pill.

Traditional antidepressant western medicine including tricyclic compounds, monoamine oxidase inhibitors and selective 5-hydroxytryptamine reuptake inhibitors, etc can effectively relieve depressive symptoms. However, many deficiencies exist such as long medication cycle, serious toxic and side effects, poor medication compliance, repeated disease delay, etc, which can greatly affect the prognosis and clinical application of the disease.^[6]^ Traditional Chinese medicine (TCM), with outstanding treatment effect on the depression-related symptoms, has been extensively applied in China to treat the depression.^[7–8]^ TCM also has therapeutic effects on depression-related symptoms. For example, Xiaoyaosan^[9]^ is used to spread liver qi, relieve depression and invigorate spleen; Kai-xin-san^[10]^ is applied to invigorate qi, nourish heart and tranquilize mind; Chaihu Shugan San,^[11]^ etc. A meta-analysis result has shown that^[12]^ Chaihu-longgu-muli decoction or combination of antidepressants is superior to antidepressants alone in the treatment of depression, which can significantly reduce the incidence of adverse reactions during medication. Jiaotai pill (JTP) was a very famous Chinese medicine formula, which was from Doctor Han Mao's medical works (Hanshi Yitong) of the Ming Dynasty about 500 years ago. JTP comprises 2 TCM herbs, Huanglian (Coptis chinensis Franch) and Rougui (Cinnamomum cassia Presl), which are mainly used to treat depression and insomnia. The dosage ratio of the 2 herbs was 10:1. The nature of Huanglian was cold while Rougui was hot. Huanglian could clear heart fire, while Rougui was used to warm kidney water based on the 5 elements of TCM. Therefore, JTP was often used to treat the disharmony of heart and kidney syndrome (See Fig. 1). Several experimental studies have demonstrated the effect of JTP on depression mouse models, but no standard clinical evidences can support those claims.^[13–16]^ Therefore, in this study, a clinical study was designed to evaluate the efficacy and safety of JTP on depression patient who was diagnosed with disharmony of heart and kidney syndrome based on the theory of TCM.

## 2 Methods/design

### 2.1 Study design

**Figure 2:**
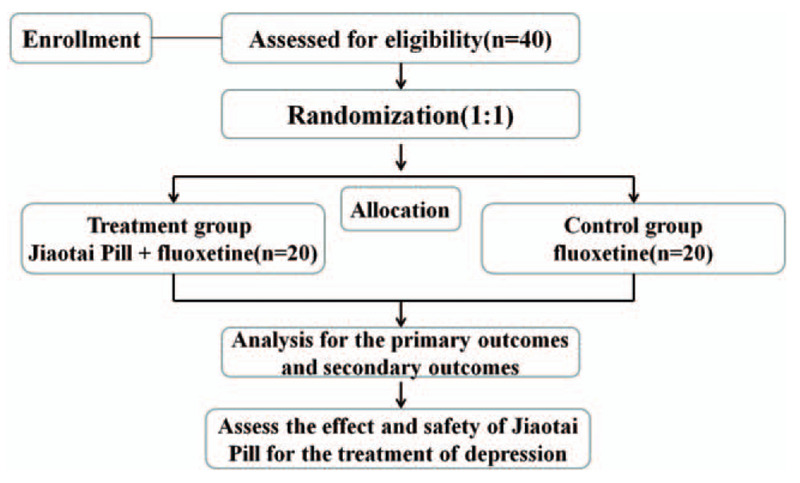
Flowchart of this study.

This was a multicenter, randomized and controlled trial to evaluate the efficacy and safety of JTP in patients with depression. A flowchart for the study was shown in Figure 2.

Qualified patients were randomly divided into the treatment and control groups with a ratio of 1:1, respectively. Recruited patients received JTP plus fluoxetine or fluoxetine alone once per day for 8 weeks. After the 8-week treatment period, the patients were then followed up for 4 weeks. The outcome measures included the Hamilton Depression (HAMD) Rating Scale scores, TCM syndrome integral scale scores, Wisconsin Card Sorting Test (WCST), blood metabonomics, urine metabonomics and brain structure and function on functional magnetic resonance imaging (fMRI).

### 2.2 Participants

#### 2.2.1 Inclusion criteria

To participate in the study, patients should meet the following criteria:

(1) Conform to depression diagnostic criteria of the Diagnostic and Statistical Manual of Mental Disorders’ American psychiatric diagnosis standard’, 4th Edition (DSM-IV);
(2) Conform to the diagnostic criteria of TCM syndrome of disharmony between the heart and kidney. The diagnostic criteria of disharmony between the heart and kidney syndrome was formulated with reference to the diagnostic standards established by the National “Eleventh Five-Year Plan” Scientific and Technological Research Project “Research on the TCM Syndrome of Depression”. The diagnosis criteria included the following items: emotional depression, insomnia and dreaming, palpitations, forgetfulness, upset, night sweating, waist and knee weakness, dizziness, dry throat and mouth, nocturnal emission, irregular menstruation, red tongue, thin and rapid pulse. Among these items, emotional depression was necessary, and 5 or more of other items were required.
(3) HAMD 24-item score between 20 and 35 points;
(4) Age between 18 and 65 years;
(5) Total Hamilton anxiety rating scale (HAMA) score of ≤21 points, depressed mood (item 6) score ≥2 points and anxious mood (item 1 of HAMA) score < 3 points;
(6) Those who had not taken antidepressants or had taken them but had stopped taking them for 2 weeks;
(7) Sign the informed consent form.

#### 2.2.2 Exclusion criteria

Patients with the following criteria would be excluded:

(1) Secondary to other mental or physical illness and depression with severe psychotic symptoms;
(2) Allergic constitution or previous research on drug allergy;
(3) Suicidal thoughts;
(4) Bipolar disorder, refractory depression;
(5) Intracranial cerebrovascular disease, neurodegenerative diseases, intracranial tumor, high blood pressure, or diabetes, leading to brain vascular distribution or abnormal blood flow of systemic disorders;
(6) Serious diseases of other systems or severe heart, liver and renal insufficiency;
(7) Glaucoma and epilepsy;
(8) Pregnancy or lactation or quasi-pregnancy;
(9) Positive urine pregnancy test in women of childbearing age;
(10) Contraindications on fMRI inspection and those who cannot complete fMRI scan or cannot adapt to the environment and noise of the fMRI machine;
(11) Participation in or planning to participate in other observational drug studies within 30 days;
(12) Poor compliance or inability to be interviewed regularly.

### 2.3 Withdrawal and discontinuation

During the trial, if they had any adverse reactions, the participants could stop the trial at any time. No strategy was adopted to improve adherence to the study drug. During the trial, the drugs related to depression were banned, while those not related to depression could be used. Patients with severe illnesses could withdraw the trial and take the appropriate treatment.

### 2.4 Ethics and recruitment

All patients signed the informed consent forms prior to inclusion, which included the research protocols, the benefits and risks, confidentiality, costs, and voluntary principles. The study has been approved by the Ethics Committee of Tianjin University of Traditional Chinese Medicine. If the protocol requires modification, ethical approval will be reapplied. Any revisions in the study protocol will be submitted to the Ethics Committee and the National Natural Science Foundation of China.

Through advertisements and referrals, a total of 40 qualified patients were recruited from 2 research centers: the First Teaching Hospital of Tianjin University of Traditional Chinese Medicine and the Tianjin Academy of Traditional Chinese Medicine Affiliated Hospital.

### 2.5 Sample size

**Figure 3:**
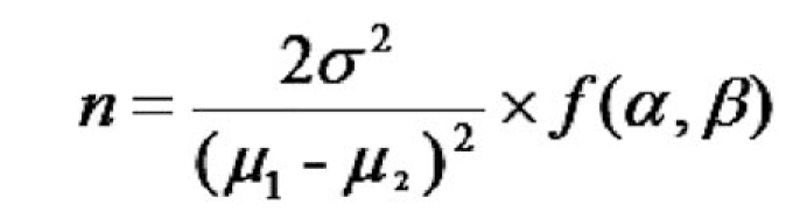
The sample size calculation formula.

In reference to the relevant literature, the specific process to estimate the sample content of each group was as follows.^[17–19]^ The sample size estimation formula for clinical trials was shown in Figure 3, where n is the number of cases required for each group, μ1 and μ2 are the expected averages of the treatment and control groups, respectively, and σ2 is the standard deviation of the control group, α = 0.05, β = 0.1, the output f(α, β) of the look-up table is 10.5. Taking the score of HAMD scale after treatment as the main evaluation index, through consulting previous literatures^[20–22]^ and taking the average, μ1 = 9.39, μ2 = 12.49, and σ2 of the control group is 2.97, the above research data were substituted into the above formula to calculate that n is 19.2, and the sample size of both the treatment and control groups was 20 cases with total 40 cases.

### 2.6 Randomization

Treatment allocation was done when participants met the inclusion criteria and signed the informed consent form. Patients were randomly divided into 2 groups, the treatment and control groups, with a 1:1 distribution ratio according to the random numbers generated by IBM SPSS Statistics software version 19.0. The recruiter obtained a sequence number from the distributor when a patient was eligible for the trial. The subjects’ allocation was concealed inside the sealed, opaque and serialized envelopes to avoid the influence from the biases of the researchers, who took responsibility of recruitment and treatment assessment. To ensure strict confidentiality, the envelopes were not transparent, even under intense light.

### 2.7 Blinding methods

This study was a double-blind trial. The doctors knew the group allocations and the treatment protocol for better operation, while the subjects and statisticians were unaware of the group allocations in order not to be disturbed by subjective factors. If emergencies occur or treatments are needed, the person responsible for the participating units will immediately report to the major investigators, and patient unblinding will be performed only with their approval. Once the allocation is unblinded, the investigators must comply with the trial requirements when analyzing and recording data.

### 2.8 Interventions

Patients randomized to the treatment group were administered one bag of JTP granules (including 15 g C.chinensis Franch and 2 g Ci.cassia Presl of crude herbs) dissolved in warm water plus fluoxetine 20 mg once daily for eight weeks. There should be at least half an hour between taking TCM and western medicine. Patients in the control group received 20 mg fluoxetine once daily for 8 weeks. JTP granules and fluoxetine were manufactured by Beijing Tcmages Pharmaceutical Co, Ltd and Eli Lilly Company, respectively.

Patients will receive trial drugs for free. The dosage of the medicine and any remnant shall be recorded, and a drug counting method will be used to monitor the adherence of patients.

### 2.9 Outcome assessment

#### 2.9.1 Primary outcomes

##### 2.9.1.1 HAMD scores

This study used 24-item HAMD scale, which is summarized as including 7 categories of factors and can reflect the patient characteristics more concisely and clearly. The seven types of depression scale factors include the following:

(1) anxiety/somatization factor, 18 points;
(2) weight: 2 points;
(3) cognitive disturbance factor, 21 points;
(4) day and night change factor, 2 points;
(5) retardation, 14 points;
(6) sleep dysfunction factor, 6 points
(7) withdrawal and self-accusation, 12 points.

Factor scores can reflect the characteristics of patients with depression symptoms as well as the characteristics of the changes before and after the drug interventions. The total scores and factor scores were compared, respectively. And both the comparison of the total scores and factor scores showed the same results that the mean scores of 4 weeks, 8 weeks, and 4 weeks after treatment were different from those of 0 week.

##### 2.9.1.2 TCM syndrome integral scale scores

TCM symptoms of all patients were evaluated by the TCM syndrome integral scale. The comparison of the total scores demonstrated that the mean scores of 4 weeks, 8 weeks, and 4 weeks after treatment were different from 0 weeks.

##### 2.9.1.3 Brain structure and function on fMRI

MRI can reveal brain tissue abnormalities in patients with depression in the aspects of brain microstructure, brain function changes and brain metabolite changes, and fMRI combines the three aspects of function, anatomy and imaging.^[23–25]^ Diffusion tensor imaging (DTI) technique was used to detect differences and changes in fractional anisotropy values in the whole brain. The changes in cerebral white matter integrity in patients with depression before and after treatment were compared in order to study the effect of JTP on brain metabolites and the changes in brain metabolites by magnetic resonance spectroscopy. The comparison of the brain structure and function on fMRI discovered that the mean scores of 8 weeks were different from 0 weeks.

#### 2.9.2 Secondary outcomes

##### 2.9.2.1 WCST

Patients were assessed for cognitive function using the computer version of WCST.WCST consists of 4 stimulation cards and 48 reaction cards, which were used to assess the cognitive function of patients by the total number of WCST, number of correct responses, total number of errors, number of persistent errors, and number of random errors. The comparison of the WCST was that the mean scores of 4 weeks, 8 weeks, and 4 weeks after treatment were different from 0 week.

##### 2.9.2.2 Blood and urine metabonomics

To systematically analyze the metabolic status of patients with depression, the metabonomics characteristics of depression and metabolic changes after treatment were studied. Metabolomics was similar with the overall concept of TCM, which accorded with the thinking of correspondence between human and nature of TCM. This technology, which showed the dynamic changes in the organism, avoiding disadvantages of using a single or a few indicators to study physiological and pathological changes, it had the characteristics of integration, dynamics, synthesis, and analysis. It had unique advantages for revealing the pathogenesis of depression and drug metabolism patterns.

#### 2.9.3 Safety reporting

Adverse drug reaction (ADR) was defined as any unfavorable and unintended sign, symptom or disease arising in participants in either group during the trial period, regardless of their relation to the experimental treatment. Appropriate measures should be adopted to ensure participants’ safety and follow up the outcome of any ADR. The relevant information for ADR was filled out in the safety evaluation form. The record included a detailed description of ADR, time of occurrence, time of suspension, duration (which can be recorded in days or hours), severity and frequency of the occurrence, manner of treatment, amount of treatment and course of treatment as well as the causal analysis of ADR and trial treatment. All clinical data for ADR were recorded in the case report forms (CRFs).

#### 2.9.4 Data collection, management, and quality control

The hard copies of CRFs were used to collect relevant data for each participant, and participants were assessed every four weeks during and after the study. Data on HAMD, WCST and TCM syndrome integral scale were collected at each visit (week 0 ± 3 days, week 4 ± 3 days, week 8 ± 3 days and week 12 ± 3 days). Blood metabolomics, urine metabolomics and fMRI were carried out only at the first and third visits (week 0 ± 3 days and week 8 ± 3 days, respectively). The process of clinical observation of the trial was shown in Table 1.

**Table 1:**
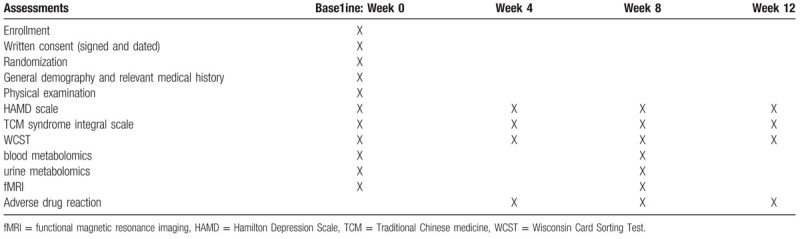
Process of clinical observation.

After being reviewed by clinical inspectors, completed CRFs were sent to a specified statistics center, and data input and processing were performed by 2 individual data administrators to ensure data accuracy. All documents gathered in this study were stored securely. Participants were referred to by a specific randomization number rather than by their names in all other documents in this study, except for the informed consent form.

A quality inspection system was developed in the treatment group, and a quality inspector was set up. The details of the quality inspector's implementation included examining the experimental protocols and procedures by the test operators and recording and reporting all research data and the truthful, accurate and complete CRFs, in order to assure that the original data were consistent.

#### 2.9.5 Data analysis

##### 2.9.5.1 Primary analysis of validity

To calculate the HAMD scores of the patients in the 2 groups during the treatment period and determine the mean and standard deviation of the values at baseline from those after treatment. The secondary analysis was described below. *Balance comparison*: To compare the demographics and other basic indicators to measure the comparability of the 2 groups. *Analysis of action mechanism:* To calculate the TCM syndrome integral scale scores, WCST, Blood and urine metabonomics, and analyze MRI of the patients in the 2 groups. Statistical analysis or Pearson correlation coefficient was performed using analysis of variance (ANOVA).

Statistical analysis was conducted as an intention-to-treat analysis by using SPSS Statistics version19.0 software. A 2-sided *P* value of <.05 was considered to be statistical significant. Baseline differences among the groups were evaluated using 1-factor ANOVA for measurement data and the *χ*^2^ test for enumeration data, respectively. In addition, *χ*^2^ test was used for categorical variables. The changes in scores from baseline to endpoint of treatment were assessed using a paired *t* test for measurement data and a signed rank test for enumeration data, respectively. Comparisons between groups were performed using ANOVA and a rank test.

The evaluation result will be analyzed with the method of intention-to-treat analysis (ITT). The ITT analysis method leads to more reliable conclusions, which prevents the cases with a poor effect in the final analysis from being excluded and therefore increases the comparability among the groups. The loss of data will be conducted according to the last observation carried forward principle.

To limit the extent of missing data, when the assigned treatment was discontinued, efforts were made to obtain the participant's consent to collect the data on treatments and outcomes.

The researchers conducting the data analysis were not involved in experimental or clinical decision-making processes to avoid any bias caused by subjective factors from the researchers.

## 3 Discussion

Depression is one of the most common mental disorders and social issues worldwide. In the modern society, the incidence of depression has risen dramatically due to an accelerated pace of life and increased work pressure.^[26]^ Treatment of depression by Western medicine has more side effects, more adverse reactions and shorter duration of action than that by TCM, therefore, Western medicine should not be used for a long term. In addition, many patients do not respond adequately to the treatment.^[27–28]^ Prevention and treatment of depression by TCM can date back thousands of years ago. TCM is more stable and effective, with fewer side effects and better patient compliance than Western medicine, these are the reasons why TCM has become a hot spot for depression in the recent years.^[29]^

JTP is a commonly used formula for clinical treatment of insomnia and depression. The research team used mouse suspension experiments and mouse forced swimming experiments to study the antidepressant effect of JTP in different proportions, the results showed that JTP had antidepressant effect, and the antidepressant effect was dose-dependent. The research group also conducted a mouse reserpine antagonism experiment, and found that JTP can exert antidepressant effects by regulating the levels of NE and 5-HT in hippocampal tissue and cortex.^[15]^ Clinically, the addition and subtraction of JTP as the main prescription can obviously relieve the symptoms of palpitations, insomnia, anxiety, and irritability in patients with depression, but no standard clinical evidences can support those claims. Therefore, we conducted a multi-center clinical randomized controlled trial, using the HAMD scale and the TCM syndrome integral scale as the evaluation criteria for the efficacy, combined with the application of multimodal fMRI technology and combining metabolomics, to analyze the changes in brain structure and brain function, blood, urine metabolic fingerprints of patients with depression, and the intervention effects of JTP, to study the efficacy and mechanism of anti-depression of JTP, and provide scientific basis for clinical treatment of depression in JTP.

Our study has several limitations. First, since this study is undertaken in China, it is uncertain whether the effects of JTP would be similar in other ethnic groups. Second, because of the small sample size, if there is sufficient funding in the future, further research will be carried out.

### 3.1 Ethical principles

This study received ethical approval from Ethics Committee of Tianjin University of Traditional Chinese Medicine (TJUTCM-EC20160004), covering all participating sites. The protocol version number is V1.00. The trial was registered at Chinese Clinical Trial Registry with registration number ChiCTR-IOR-17010748. It was stated that all items from the WHO Trial Registration Data Set could be found within the protocol. Important changes to the protocol will be submitted to the TJUTCM for review. Informed consent will be obtained from all participants in the trial. The trial is currently recruiting patients.

## Acknowledgments

We gratefully acknowledged the cooperation of all research staff and participants.

## Author contributions

Zhihuan Zhou conceived and designed the trial and wrote the manuscript draft. Chunquan Yu conceived the study and performed the critical revision of the manuscript. Shufei Fu was the general supervisor for this research and wrote the manuscript draft. Yijia Liu drafted the manuscript. Huaien Bu was responsible for the statistical design and helped in the design of the study. Yuhan Wang, Yan Mei and Yi Tong were in charge of the recruitment of patients, data collection and management. All authors read and approved the final manuscript.

## References

[1] Brown GK Karlin BE Trockel M Effectiveness of cognitive behavioral therapy for veterans with depression and suicidal ideation. Arch Suicide Res 2016;20:677–82. 26983897 10.1080/13811118.2016.1162238

[2] Orzechowska A Filip M Galecki P Influence of pharmacotherapy on cognitive functions in depression: a review of the Literature. Med Sci Monit 2015;21:3643–51. 26599597 10.12659/MSM.895156 PMC4664223

[3] Uher R Pavlova B Long-term effects of depression treatment. Lancet Psychiatry 2016;3:95–6. 26777772 10.1016/S2215-0366(15)00578-7

[4] Smith K Mental health: a word of depression. Nature 2014;515:181. 25391942 10.1038/515180a

[5] Lasserre AM Marti-Soler H Strippoli MP Clinical and course characteristics of depression and all-cause mortality: a prospective population-based study. J Affect Disord 2016;189:17–24. 26402343 10.1016/j.jad.2015.09.010

[6] Yu Dongshan Gao Zhenzhong Handbook of Rational Drug Use in Psychiatry. 2005;Nanjing: Jiangsu Science and Technology Press, [Artical in Chinese].

[7] Bablis P Pollard H Anxiety and depression profile of 188 consecutive new patients presenting to a Neuro-Emotional Technique practitioner. J Altern Complement Med 2009;15:121–7. 19236170 10.1089/acm.2007.0805

[8] Wang XJ Li J Zou QD Wuling Capsule for climacteric patients with depression and anxiety state: a randomized, positive parallel controlled trial. Zhong Xi Yi Jie He Xue Bao 2009;7:1042–6. [Artical in Chinese]. 19912735 10.3736/jcim20091104

[9] Liu CC Wu YF Feng GM Plasma-metabolite-biomarkers for the therapeutic response in depressed patients by the traditional Chinese medicine formula Xiaoyaosan: A 1 H NMR-based metabolomics approach. J Affect Disord 2015;185:156–63. 26186531 10.1016/j.jad.2015.05.005

[10] Zhu Y Chao C Duan X Kai-Xin-San series formulae alleviate depressive-like behaviors on chronic mild stressed mice via regulating neurotrophic factor system on hippocampus. Sci Rep 2017;7:1–5. 28469194 10.1038/s41598-017-01561-2 PMC5431115

[11] Zhang YJ Huang X Wang Y Ferulic acid-induced anti-depression and prokinetics similar to Chaihu-Shugan-San via polypharmacology. Brain Res Bull 2011;86:222–8. 21791239 10.1016/j.brainresbull.2011.07.002

[12] Gao Li Jia Chunhua Ma Cui Lan Meta-analysis of chaihu plus longgu muli decoction for depression. Henan Tradit Chin Med 2018;38:206–10. [Artical in Chinese].

[13] Zhang M Yu CQ Research progress on antidepressant effects of Jiaotai pills. Tianjin J Tradit Chin Med 2012;29:101–4. [Artical in Chinese].

[14] Zhou ZH Gao S Li L Clinical and pharmacological research summary of Jiaotai pills. Tianjin J Tradit Chin Med 2014;03:190–2. [Artical in Chinese].

[15] Yang S Pan Y Song YQ Effects of Jiaotai pill on behavior and monoamine neurotransmitters of depression rat model. Chin Tradit Herb Drugs 2016;47:4218–23. [Artical in Chinese].

[16] Jing HR Experience of Zhou Shaohua in the treatment of depression. Liaoning J Tradit Chin Med 2009;36:1660–2. [Artical in Chinese].

[17] Sakpal T Sample size estimation in clinical trial. Perspect Clin Res 2010;1:67–9. 21829786 PMC3148614

[18] Jingpu S Estimation method of sample size in clinical research. Chin Clin Rehabil 2003;7:1569–71. [Artical in Chinese].

[19] Wu X Li CJ Ding BF Sample size estimation in superiority clinical trials for two means comparison. J Math Med 2013;26:517–9. [Artical in Chinese].

[20] Yan JM Zhou HP Li JL Clinical study of Jieyu Anshen Dingzhi decoction combined with paroxetine treating depression. World Chin Med 2014;9:1178–80. [Artical in Chinese].

[21] Huang SH Chen LQ Liu ZJ Clinical observation on Jieyu Jiaonang combined with paroxetine treating 50 depression cases. J Tradit Chin Med 2015;56:778–81Ob. [Artical in Chinese].

[22] Zhang J Observation of clinical effect of Carefree pill combined sertraline treatment of melancholia. China Med Pharm 2015;5:75–9. [Artical in Chinese].

[23] Mattson WI Hyde LW Shaw DS Clinical neuroprediction: amygdala reactivity predicts depressive symptoms 2 years later. Soc Cogn Affect Neurosci 2016;11:892–8. 26865423 10.1093/scan/nsw018 PMC4884317

[24] Opmeer EM Kortekaas R van Tol MJ Changes in regional brain activation related to depressive state: a 2-year longitudinal functional MRI study. Depress Anxiety 2016;33:35–44. 26378742 10.1002/da.22425

[25] Rayner G Jackson G Wilson S Cognition-related brain networks underpin the symptoms of unipolar depression: evidence from a systematic review. Neurosci Biobehav Rev 2016;61:53–65. 26562681 10.1016/j.neubiorev.2015.09.022

[26] Pytka K Dziubina A Młyniec K The role of glutamatergic, GABA-ergic, and cholinergic receptors in depression and antidepressant-like effect. Pharmacol Rep 2016;68:443–50. 26922551 10.1016/j.pharep.2015.10.006

[27] Muñoz RF Bunge EL Prevention of depression worldwide: a wake-up call. Lancet Psychiatry 2016;3:306–7. 26827251 10.1016/S2215-0366(15)00555-6

[28] Schipper S High prevalence of depression in medical residents: the sad reality of medical training. Evid Based Med 2016;21:118. 26940711 10.1136/ebmed-2016-110381

[29] Gao MC Li J Qi YY Syndrome differentiation of traditional chinese medicine treatment of depression in 276 cases. J Pract Tradit Chin Intern Med 2013;27:80–1. [Artical in Chinese].

